# Interplay between autotrophic and heterotrophic prokaryotic metabolism in the bathypelagic realm revealed by metatranscriptomic analyses

**DOI:** 10.1186/s40168-023-01688-7

**Published:** 2023-11-04

**Authors:** Abhishek Srivastava, Daniele De Corte, Juan A. L. Garcia, Brandon K. Swan, Ramunas Stepanauskas, Gerhard J. Herndl, Eva Sintes

**Affiliations:** 1 Department of Functional and Evolutionary Ecology, Bio-Oceanography and Marine Biology Unit, University of Vienna, Djerassiplatz 1, 1030 Vienna, Austria; 2 Konrad Lorenz Institute of Ethology, University of Veterinary Medicine Vienna, Savoyenstrasse 1a, 1160 Vienna, Austria; 3 Institute for Chemistry and Biology of the Marine Environment, Carl Von Ossietzky University, Oldenburg, Germany; 4 Currently at Ocean Technology and Engineering Department, National Oceanography Centre, Southampton, UK; 5 Department of Informatics, INS La Ferreria, 08110 Montcada i Reixach, Spain; 6 National Biodefense Analysis and Countermeasures Center, Frederick, MD 21702 USA; 7 Bigelow Laboratory for Ocean Sciences, East Boothbay, ME 04544 USA; 8 Department of Marine Microbiology and Biogeochemistry, NIOZ, Royal Netherlands Institute for Sea Research, AB Den Burg, The Netherlands; 9 Ecosystem Oceanography Group (GRECO), Instituto Español de Oceanografía (IEO-CSIC), Centro Oceanográfico de Baleares, Palma, Spain

**Keywords:** Metatranscriptome, Gene expression, Bathypelagic, Labrador Sea Water, Thiosulfate, Dissolved organic matter (DOM), Dissolved inorganic carbon (DIC) fixation, Chemoautotrophy, Heterotrophy

## Abstract

**Background:**

Heterotrophic microbes inhabiting the dark ocean largely depend on the settling of organic matter from the sunlit ocean. However, this sinking of organic materials is insufficient to cover their demand for energy and alternative sources such as chemoautotrophy have been proposed. Reduced sulfur compounds, such as thiosulfate, are a potential energy source for both auto- and heterotrophic marine prokaryotes.

**Methods:**

Seawater samples were collected from Labrador Sea Water (LSW, ~ 2000 m depth) in the North Atlantic and incubated in the dark at in situ temperature unamended, amended with 1 µM thiosulfate, or with 1 µM thiosulfate plus 10 µM glucose and 10 µM acetate (thiosulfate plus dissolved organic matter, DOM). Inorganic carbon fixation was measured in the different treatments and samples for metatranscriptomic analyses were collected after 1 h and 72 h of incubation.

**Results:**

Amendment of LSW with thiosulfate and thiosulfate plus DOM enhanced prokaryotic inorganic carbon fixation. The energy generated via chemoautotrophy and heterotrophy in the amended prokaryotic communities was used for the biosynthesis of glycogen and phospholipids as storage molecules. The addition of thiosulfate stimulated unclassified bacteria, sulfur-oxidizing Deltaproteobacteria (SAR324 cluster bacteria), Epsilonproteobacteria (*Sulfurimonas* sp.), and Gammaproteobacteria (SUP05 cluster bacteria), whereas, the amendment with thiosulfate plus DOM stimulated typically copiotrophic Gammaproteobacteria (closely related to *Vibrio* sp. and *Pseudoalteromonas* sp.).

**Conclusions:**

The gene expression pattern of thiosulfate utilizing microbes specifically of genes involved in energy production via sulfur oxidation and coupled to CO_2_ fixation pathways coincided with the change in the transcriptional profile of the heterotrophic prokaryotic community (genes involved in promoting energy storage), suggesting a fine-tuned metabolic interplay between chemoautotrophic and heterotrophic microbes in the dark ocean.

Video Abstract

**Supplementary Information:**

The online version contains supplementary material available at 10.1186/s40168-023-01688-7.

## Background

The dark ocean (below 200 m depth) harbors approximately 75% and 50% of the global ocean’s prokaryotic biomass and production, respectively [1]. Prokaryotes inhabiting the dark ocean greatly depend for their growth on the fluxes of particulate organic matter (POM) generated in the sunlit surface ocean [2, 3]. However, the mismatch between current estimates of POM exported from the sunlit surface layers and the bacterial carbon demand in the dark ocean suggests the presence of alternative sources of energy and carbon for dark ocean prokaryotes [1, 2]. Dissolved inorganic carbon (DIC) fixation rate measurements [4], isotopic [5], and molecular approaches [6, 7] have contributed to the increasing evidence of chemoautotrophy as a significant source of organic matter production in the dark ocean.

However, the energy sources fuelling chemoautotrophy remain enigmatic. Inorganic reduced nitrogen and sulfur compounds, methane or hydrogen [6–9] are potential inorganic energy sources. Among the numerous reduced sulfur compounds, thiosulfate is ubiquitously present throughout the ocean water column, albeit at low concentrations [10]. Thiosulfate can be generated from the oxidation of other inorganic sulfur compounds or as a by-product from oxidation or fermentation of organosulfonates like taurine and other organic sulfur compounds by aerobic and anaerobic bacteria [11, 12].

Photoautotrophs, chemoautotrophs, and even heterotrophs can utilize reduced sulfur compounds as energy source mainly through the sulfur-oxidizing (sox) enzyme system [13, 14]. Chemolithoautotrophy based on thiosulfate utilization has been reported in axenic aquatic bacterial cultures [15, 16], in marine mixotrophic sulfur-oxidizing bacteria UBA868 [17], and in hydrothermal vent plumes [9]. Conversely, an earlier transcriptomic study on mesopelagic prokaryotic communities revealed no clear effects of thiosulfate on chemoautotrophs [18]. Enhanced organic carbon assimilation coupled with oxidation of thiosulfate and reduced respiration has been reported in marine heterotrophic bacteria [19]. This latter process might be particularly relevant in nutrient-rich microenvironments, such as marine snow, sinking macro-organism carcasses, or fecal pellets [20, 21]. However, the potential role of thiosulfate as a source of energy in the oxygenated bathypelagic realm and the relevant taxa capable of reduced sulfur-based chemoautotrophy remain largely unknown.

In the present study, we performed measurements of DIC fixation and community transcriptomic analyses in thiosulfate amended and unamended bathypelagic waters to elucidate the potential of thiosulfate and thiosulfate combined with dissolved organic matter (DOM) in chemoautotrophic and heterotrophic metabolism.

## Methods

### Sample collection and DIC fixation rate measurements

Seawater was collected during the MOCA cruise with RV *Pelagia* from 2000 m depth in the North Atlantic (Figure S1) in July 2012 using 12 L Niskin bottles mounted on a CTD rosette sampler with sensors for conductivity–temperature–depth (CTD), salinity, oxygen, fluorescence, and optical backscattering. Based on corresponding salinity and temperature characteristics, the collected seawater was identified as Labrador Sea Water (Table S1). Dissolved inorganic nutrient concentrations (NO_3_^−^, NO_2_^−^, PO_4_^3−^) were determined on 0.2 μm filtered seawater in a TRAACS 800 autoanalyzer system immediately after collecting the samples following established protocols [22]. Microbial leucine incorporation rates were measured as previously described [4]. Briefly, 50 mL triplicate seawater samples were inoculated with 10 nM [^3^H]-leucine (final concentration) and incubated in the dark at in situ temperature for 4–7 h together with triplicate formaldehyde-killed controls. To terminate the incubation, formaldehyde (2% final concentration) was added to the samples, and the samples and controls were filtered onto 0.2 µm polycarbonate filters. Subsequently, the filters were rinsed with 5% ice-cold trichloroacetic acid. The dry filters were placed in scintillation vials with 8 mL scintillation cocktail (FilterCount, Canberra-Packard), and after about 18 h counted on board the research vessel in a liquid scintillation counter (LKB Wallac). The instrument was calibrated with internal and external standards. The water was split in duplicate 2 L acid-cleaned polycarbonate bottles and amended with 10 µM ammonia, 1 µM sulfite (Na_2_SO_3_), 1 µM thiosulfate (Na_2_S_2_O_3_), 10 µM of each glucose and acetate (DOM), or 1 µM thiosulfate and 10 µM of each glucose and acetate (thiosulfate + DOM treatment). All the treatments together with an unamended control were incubated at in situ temperature in the dark for 1 h or 72 h. At the end of the incubation, DIC fixation was measured via ^14^C-bicarbonate uptake as previously described [4]. Briefly, 50 mL triplicate water samples and duplicate formaldehyde-killed controls were incubated in the dark at in situ temperature after the addition of 100 µCi of ^14^C-bicarbonate for 60–72 h [4]. Subsequently, the incubations were terminated by formaldehyde addition (2% final concentration) to the samples. Samples and killed controls were filtered onto 0.2 μm polycarbonate filters and rinsed with filtered seawater (< 0.2 µm). Subsequently, the filters were fumed with concentrated HCl for 12 h. Filters were placed in scintillation vials together with 8 mL of scintillation cocktail (FilterCount, Canberra-Packard). After about 18 h, the samples were counted on board in the liquid scintillation counter (LKB Wallac). The instrument was calibrated with internal and external standards. Prokaryotic abundance (Table S1) was measured by flow cytometry following the protocol described by Sintes et al. [23], and dissolved organic carbon (DOC) concentration (Table S1) was determined in triplicate GF/F filtered water samples using a Shimadzu TOC-5000 analyzer [24, 25].

### RNA extraction, cDNA library construction, and sequencing

Two liters of water from the original sample were filtered immediately after collection onto 0.2 µm pore-size SUPOR filters (Pall), and stored in cryovials at − 80 ºC after the addition of 1 mL RNAlater. One replicate from the different treatments was filtered 1 h after the addition of the different substrates (T1), while the second replicate was filtered 72 h after the addition of substrates (T72). The filtration took always < 15 min and the filters were stored as previously described for the original sample. Therefore, it should be noted that only one replicate per treatment per time point is available in this study, which limits the possibility of statistically analyzing and disentangling technical vs. true variability [26].

RNA was extracted from the original sample, the unamended control, the thiosulfate, and the thiosulfate + DOM treatments after 1 h and 72 h of incubation. The filters were thawed on ice and the RNAlater (Ambion, USA) was pipetted and concentrated to ~ 40–60 µL by centrifugation using Amicon Ultracel 2 mL 10 KDa centrifugal filters (Millipore Corp., USA), at 4000 × *g* for 45 min (4 ºC). Thawed filters were cut into small pieces using sterile razor blades and placed inside clean 2 mL screwcap tubes. RNA was extracted from the filter pieces and the concentrated RNAlater solution using the mirVana™ miRNA Isolation Kit (Ambion, USA). Approximately, 200 µL of a 1:1 zirconia/silica bead (BioSpec Products, USA) solution was added to each 2 mL tube prior to the cell lysis step. DNA was removed with the TURBO DNA-freeTM Kit (Ambion, USA), and the total RNA extracted was purified and concentrated with the RNeasy MinElute Cleanup Kit (Qiagen, USA). The absence of DNA from extracted RNA was verified by 40 cycles of PCR amplification of partial 16S rRNA genes using the primers 27F and 907R [27]. RNA quality was checked using the Agilent 2200 TapeStation system.

cDNA libraries were generated using the Ovation RNA-Seq System V2 Kit (NuGEN Technologies, USA), purified using the RNeasy MinElute Cleanup Kit (Qiagen, USA), and quantified with a Qubit™ 3.0 Fluorometer (ThermoFisher Scientific, USA). Sequencing libraries were constructed using Illumina’s Truseq RNA Sample Prep kit v2 according to the manufacturer’s instructions and paired-end sequenced using a NextSeq 500 (Illumina) in 2 × 150 bp mode using v.1 reagents at the Bigelow Laboratory Single Cell Genomics Center (https://scgc.bigelow.org).

### Bioinformatic analyses

Adapter sequences were removed with Trimmomatic [28] and paired-end sequences were merged with PEAR using default settings [29]. The resulting paired-end sequence quality was assessed using PRINSEQ lite 0.20.3 [30], and all sequences with these characteristics were removed: sequences < 100 bp in length and a mean quality score < 30, sequences containing any ambiguities (Ns), sequences with poly-A and poly-T runs of at least 5 bp, all forms of replicate and duplicate sequences, and sequences with a minimum entropy value of 70. Ribosomal RNA sequences were removed prior to downstream analysis with riboPicker [31]. To estimate taxon and transcript abundances, the reads from the seven samples were used to query against KEGG GENES protein database using the DIAMOND BLASTX [32] with an e-value cutoff of 10^−5^ and a minimum alignment length cutoff of 30 amino acids. The resulting reads were subsequently screened for taxonomic classification against the NCBI non-redundant protein database using KAIJU [33] with MEM mode. Taxonomic classification and functional annotation were analyzed in R. However, it should be mentioned that the lack of corresponding metagenomes from the same sample might hinder taxonomic identification due to the limitations of publicly available genome reference databases. The transcript abundance was normalized by the total read abundance (transcript per million reads) for each sample. Subsequently, *Z*-score scaling was applied to show the number of standard deviations above and below the mean transcript expression signal in each sample. *Z*-score scaling was calculated as* Z*-score = (value–mean (transcript)/standard deviation (transcript).

## Results and discussion

### Potential energy sources supporting chemoautotrophy in the deep ocean

Chemoautotrophy represents a fresh source of organic carbon for dark ocean prokaryotes [2], and potentially contributes between 12 and 72% to the heterotrophic prokaryotic carbon demand in mesopelagic waters [34]. Although the energy sources necessary for chemolithoautotrophy, i.e., reduced inorganic compounds, are technically difficult to measure in the well-oxygenated deep ocean, growing evidence based on –omics and other molecular techniques points to the potential use of nitrite [7], ammonia [8], hydrogen [9], and reduced sulfur compounds [6] by dark ocean prokaryotes.

**Fig. 1:**
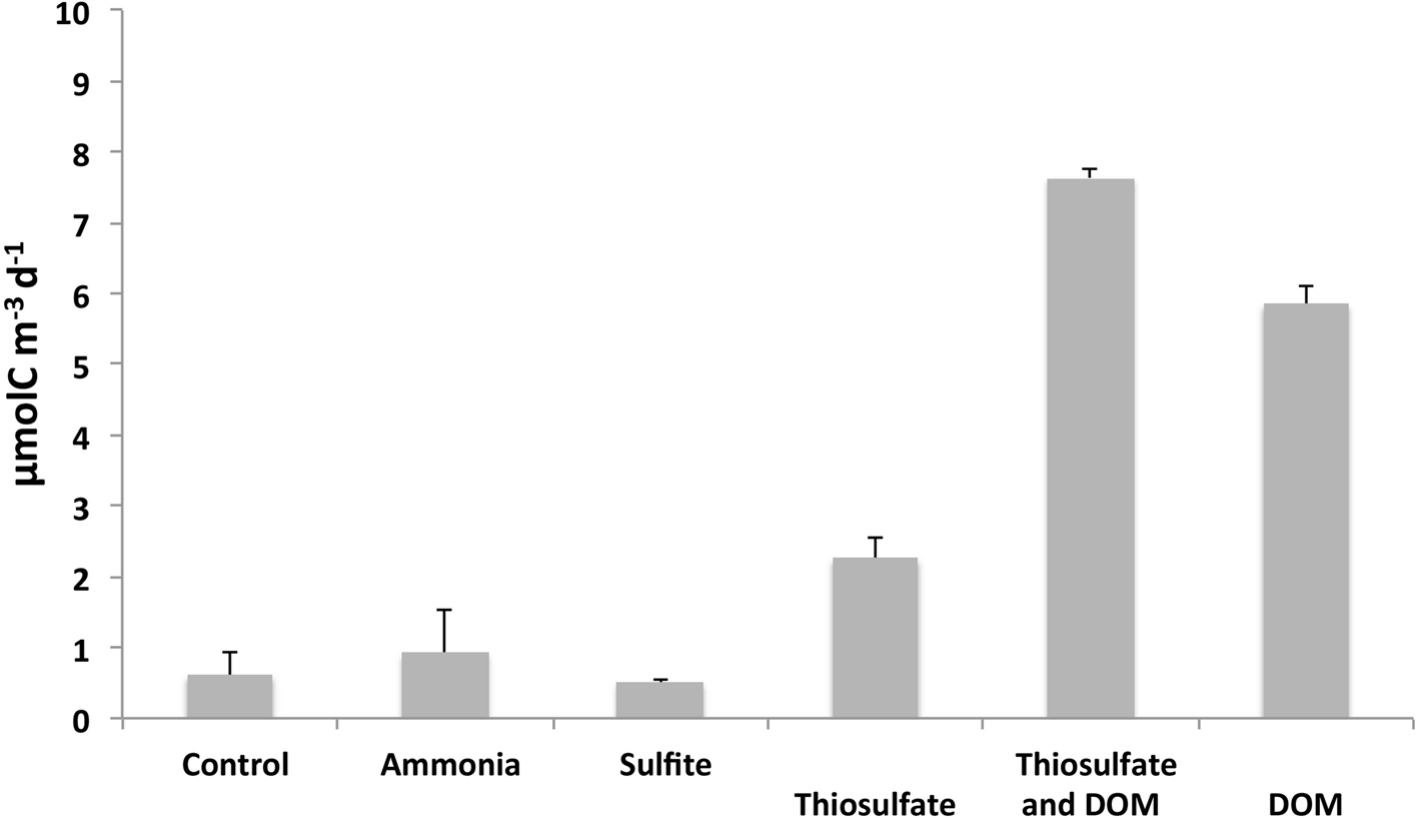
Dissolved inorganic carbon (DIC) fixation in the bathypelagic prokaryotic community amended with ammonia (10 µM), sulfite (1 µM), thiosulfate (1 µM), thiosulfate + DOM (1 µM thiosulfate + 10 µM glucose + 10 µM acetate), DOM (10 µM glucose + 10 µM acetate), and the unamended control (control) after 72 h incubation. The bars represent the average ± standard error of three replicate bottles

A natural prokaryotic community collected from 2000 m depth in the North Atlantic (Figure S1), representing Labrador Sea Water (Table S1), was amended with various potential substrates for heterotrophic and chemoautotrophic prokaryotes (see Material and Methods for details). Ammonium and sulfite did not result in significant stimulation of prokaryotic dissolved inorganic carbon (DIC) fixation (Fig. 1) as compared to a control community without any amendments (*t* test, *p* > 0.49 and *p* > 0.63, respectively). However, the thiosulfate amendment resulted in an approximately fourfold (2.2 µmol C m^−3^ d^−1^) increase in DIC fixation compared to the unamended control treatment (0.6 µmol C m^−3^ d^−1^) (Fig. 1) (see “Material and methods” section for a detailed description of the experimental set-up). Thus, the response of bathypelagic prokaryotes to potential energy sources for chemoautotrophy seems to differ from that of mesopelagic communities, which did not respond with elevated activity upon thiosulfate amendment [18]. DIC fixation also increased in response to dissolved organic matter (DOM) amendment by ~ tenfold, as was previously shown for mesopelagic prokaryotes [18], whereas a 12-fold (7.6 µmol C m^−3^ d^−1^) DIC fixation was observed in the thiosulfate plus DOM amendment compared to the control treatment (Fig. 1). Thiosulfate is one of the most ubiquitously distributed inorganic reduced sulfur compounds in the ocean [10], and can potentially be used as sulfur [9] or energy source for autotrophic [35] and heterotrophic [36] processes. The rates measured in the different treatments were within the range of rates previously reported for the dark ocean [4] and indicate the potential of thiosulfate as an energy source for chemoautotrophy. However, the larger increase in DIC fixation rates in the thiosulfate plus DOM addition as compared to the addition of each compound separately suggests a synergy and interconnectivity between autotrophic and heterotrophic metabolic processes and/or increased anaplerotic reactions under energy-replete conditions.

### Transcriptional response of bathypelagic prokaryotes to thiosulfate

**Fig. 2:**
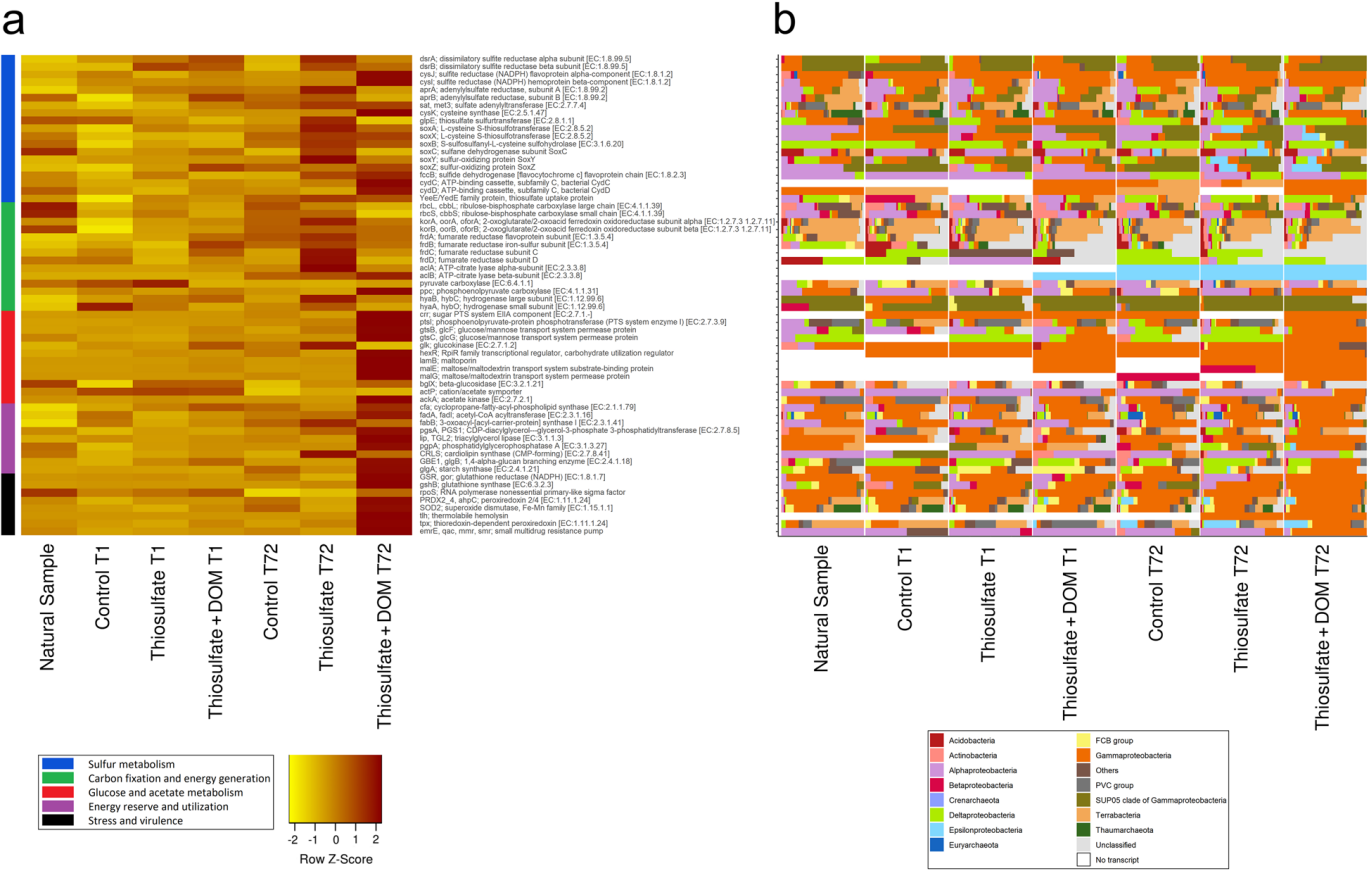
Heatmap representing normalized transcript abundance (transcript per million reads) of selected genes from bathypelagic prokaryotic communities in the different treatments and the original community (**A**). Light yellow to dark brown color range represents the increment in transcript abundance. Prokaryotic taxa affiliation of different gene transcripts (**B**). White bars indicate the absence of the corresponding gene. Natural sample stands for natural communities from Labrador Sea Water. Control T1 and Control T72 indicate prokaryotic communities filtered after 1 h and 72 h incubation, respectively. Thiosulfate T1 and Thiosulfate T72 indicate thiosulfate treated prokaryotic communities filtered after 1 h and 72 h incubation, respectively. Thiosulfate + DOM T1 and Thiosulfate + DOM T72 indicate thiosulfate + DOM (1 µM thiosulfate + 10 µM glucose + 10 µM acetate) treated prokaryotic communities filtered after 1 h and 72 h incubation, respectively

Thiosulfate can be used as a source of energy or sulfur for prokaryotes, which can oxidize, reduce, or disproportionate it [37]. Thiosulfate oxidation can generate up to eight electrons that may participate in the electron transport chain for energy generation. Prokaryotic thiosulfate oxidation to sulfate, and further reduction to sulfide, is modulated by the sulfur-oxidizing (Sox) enzymes, ATP-sulfurylase (SAT), APS reductase (APR), and dissimilatory sulfite reductase (DSR) [38, 39]. We observed that the thiosulfate amendment stimulated microbial *sox* genes expression up to twofold after 72 h in comparison to the unamended community (Fig. 2, Table S2). *sox* genes (*soxABCXYZ*) were taxonomically mainly assigned to Gammaproteobacteria (17–65%), Deltaproteobacteria (4–55%), Alphaproteobacteria (14–30%), Epsilonproteobacteria (7–24%), and unclassified (5–19%) members (Fig. 2, Table S3). Transcripts of dissimilatory sulfite reductase (*dsr*) subunits responsible for sulfite reduction were 2- to threefold upregulated in thiosulfate-amended prokaryotic communities (Fig. 2, Table S2). Another interesting observation was the increase by up to 3.6-fold of oxygen-tolerant hydrogenase subunit transcripts (*hyaA* and *hyaB*) in the thiosulfate-amended samples as compared to the control, indicating that oxidation of hydrogen may also contribute to the energy generation in the deep ocean (Table S2). Sulfur-oxidizing SUP05 bacteria perform hydrogen oxidation in hydrothermal vent plumes [9], and hydrogenase genes are widespread in diverse marine bacterial clades [40, 41]. In our treatments, hydrogenase gene transcripts were putatively affiliated with the SUP05 clade of marine Gammaproteobacteria *Candidatus* Thioglobus sp. (Fig. 2, Table S3). This finding points to a widespread plasticity of the chemoautotrophs in the dark ocean, which may utilize different electron donors and acceptors depending on the environmental availability.

Thiosulfate amendment resulted in the stimulation of transcription of genes involved in the reductive citric acid cycle (ATP citrate lyase (*acl*), 2-oxoglutarate:ferredoxin oxidoreductase (*ogor*), fumarate reductase (*frd*)) as a possible pathway for chemoautotrophy (up to sixfold compared to the control treatment), whereas transcript abundance related to anaplerotic carbon-fixation pathways (phosphoenolpyruvate carboxylase (*pepc*)) was slightly lower (0.7-fold) than in the unamended control (Fig. 2, Table S2). Members of the Epsilonproteobacteria have previously been shown to utilize thiosulfate and fix DIC by using the reductive TCA cycle [42, 43]. The frd complex is located in the cytoplasmic membrane and, to maintain the lipid-to-protein ratio, the cell synthesizes phospholipid cardiolipin [44, 45]. Cardiolipin has a conical structure that relaxes or stabilizes the negative curvature regions of membrane structures [46]. Overexpressed fumarate reductase complex is located at the cytoplasmic membrane along with other membrane-associated proteins. Consequently, lipids must also increase to maintain a constant lipid/protein ratio of the membrane, resulting in higher expression of cardiolipin [44, 45]. These findings support the upregulation of cardiolipin synthase-encoding genes in the thiosulfate amended treatment by up to 2.5-fold compared to the control treatment (Fig. 2, Table S2). Thiosulfate amendment did not stimulate anaplerotic carboxylation pathways, e.g., pyruvate carboxylase (*pc*) and phosphoenolpyruvate carboxylase (*pepc*) transcript abundances were similar in the amended and unamended treatments (Fig. 2).

Reports on chemoautotrophy by members of the SUP05 cluster and UBA868 in marine environments suggest the coupling of the Calvin-Benson cycle and thiosulfate oxidation to fix DIC [9, 17, 47]. However, although Calvin cycle genes were detected, they were not upregulated in the amended treatment during this study (Fig. 2, Table S2). Up to 35% of all *rubisco* transcripts were assigned to unclassified bacteria followed by Gammaproteobacteria (~ 25%) and unclassified Actinobacteria (14%) (Table S3). Several of these taxa have been previously reported to harbor *rubisco* and *sox* genes and conduct dark DIC fixation in the deep Atlantic Ocean [6]. The Calvin cycle and *sox* gene expression were possibly harbored within the same microorganism in an uncultured SUP05 cluster bacterium, whereas reductive TCA genes were possibly coupled to *sox* gene expression in Epsilonproteobacteria, such as *Sulfurimonas* sp. (Table S3).

### Synergy between thiosulfate and DOM on prokaryotic activity

The effect of the addition of thiosulfate plus DOM on the DIC fixation was larger than either thiosulfate or DOM separately (Fig. 1). The combined effect of reduced sulfur compounds and DOM might be particularly relevant in nutrient-enriched pelagic microenvironments such as marine snow or microenvironments associated to organisms such as zooplankton [21, 48]. DOM has been shown to stimulate anaplerotic DIC fixation in natural mesopelagic communities [18]. Thiosulfate can provide additional energy to different marine heterotrophic prokaryotes as an auxiliary electron donor to tetrathionate, promoting the use of available organic matter for biosynthesis rather than respiration [19, 49]. Changes in bacterial abundance especially in the prokaryotic activity after sulfur and/or carbon amendment possibly have contributed to the overexpression of the pathways involved in their metabolism and the energy funneled in the bacterial communities studied here.

The combined addition of thiosulfate plus DOM (glucose + acetate) to bathypelagic communities resulted in a 3.5-fold increase of phosphoenolpyruvate carboxylase (*pepc*)-transcripts as compared to the unamended community, suggesting a significant contribution of anaplerotic reactions to dark DIC fixation by bathypelagic prokaryotes when organic carbon is available (Fig. 2, Table S2). The majority of *pepc* transcripts were phylogenetically associated with members of the Gammaproteobacteria (~ 88%) (Table S3). Heterotrophic prokaryotic DIC assimilation via anaplerotic reactions can range between 1 and 15% of the carbon incorporation by heterotrophic prokaryotes [50–52] and has been suggested as a strategy to compensate for metabolic imbalances under oligotrophic [53] and nutrient-depleted conditions [54]. However, heterotrophic DIC fixation also increases with increasing heterotrophic activity [18, 55] and organic matter availability [50–52] in agreement with our findings.

Gene expression of sox enzymes slightly increased (by ~ 1.3-fold) in the community amended with thiosulfate + DOM as compared to the unamended microbial community after 72 h (Fig. 2, Table S2). Transcripts of dissimilatory sulfite reductase (*dsr*) subunits were ~ twofold upregulated in thiosulfate + DOM as compared to the control treatment (Fig. 2, Table S2). Thus, *sox* and *dsr* showed a higher stimulation of gene expression in the thiosulfate than in the thiosulfate + DOM treatment (Fig. 2, Table S2).

Sulfur-oxidizing Epsilonbacteria (such as *Sulfurimonas gotlandica*) was reported to yield higher cell abundances when amended with reduced inorganic sulfur compounds as compared to organic matter (acetate, pyruvate, or peptone) [56] supporting our finding of higher relative abundances of *sox* gene transcripts in the thiosulfate than in the thiosulfate + DOM treatment (see above). This indicates that sulfur oxidizers may not be key players in the DOM catabolism in the deep ocean. However, such labile DOM is not available in the deep ocean and thus, this interpretation requires further investigation as sulfur oxidizers might play a role in catabolizing recalcitrant compounds, as it has been shown with hydrocarbons [57]. Glucose-specific phospho-transferase system/transporter-encoding genes were upregulated (more than 130-fold) and down-regulated in the thiosulfate + DOM and thiosulfate amended communities, respectively (Fig. 2, Table S2). The phospho-transferase system is responsible for the import of glucose and for phosphorylation of glucose molecules [58]. Taken together, the down-regulation of *glk* (glucokinase, K00845) and upregulation of *hexR* gene (K19337), encoding for a *glk* gene repressor (Fig. 2, Table S2), suggests a genetic mechanism to regulate glucose utilization within the cell [59] when excess substrate is available, such as in the thiosulfate + DOM amended community. This glucose transporter was putatively assigned to *Vibrio* sp. and *Moritella* sp. (Table S3) based on amino acid homology identity (BLAST-P)), and the HexR was assigned to Vibrionales and Alteromonadales (Table S3). Additionally, glucose and maltose transporter-encoding genes (*gtsABC* and *malEFGK*) and amino acid transporter-encoding genes (*aapJQMP* and *aotJMQP*) were also upregulated in the thiosulfate + DOM treatment (Fig. 2, Table S2).

Acetate permease (*actP*) and acetate kinase (*ackA*) transcripts were upregulated (1.2- and sevenfold, respectively) in the thiosulfate + DOM amended communities compared to the control (Fig. 2, Table S2). The upregulation of *actP* and *ackA* coincided with the upregulation of cysteine synthase (*cysK*) by approximately threefold (Fig. 2, Table S2). *cysK* is responsible for the biosynthesis of cysteine and results in the intracellular production of acetate as a by-product [60]. Interestingly, *ackA* transcripts were similar or slightly down-regulated (0.8-fold) in the thiosulfate amended treatment compared to the control (Fig. 2, Table S2). Thus, the higher transcript abundances of the *ackA* gene in the thiosulfate + DOM than in the thiosulfate treatment suggest a control of the utilization of excess acetate via phosphorylation that could otherwise lead to an accumulation of intracellular acetate. An excess of glucose together with the inability to metabolize glycolytic products and acetate may lead to a carbon overflow and even cell death associated with intracellular acidification [61].

**Fig. 3:**
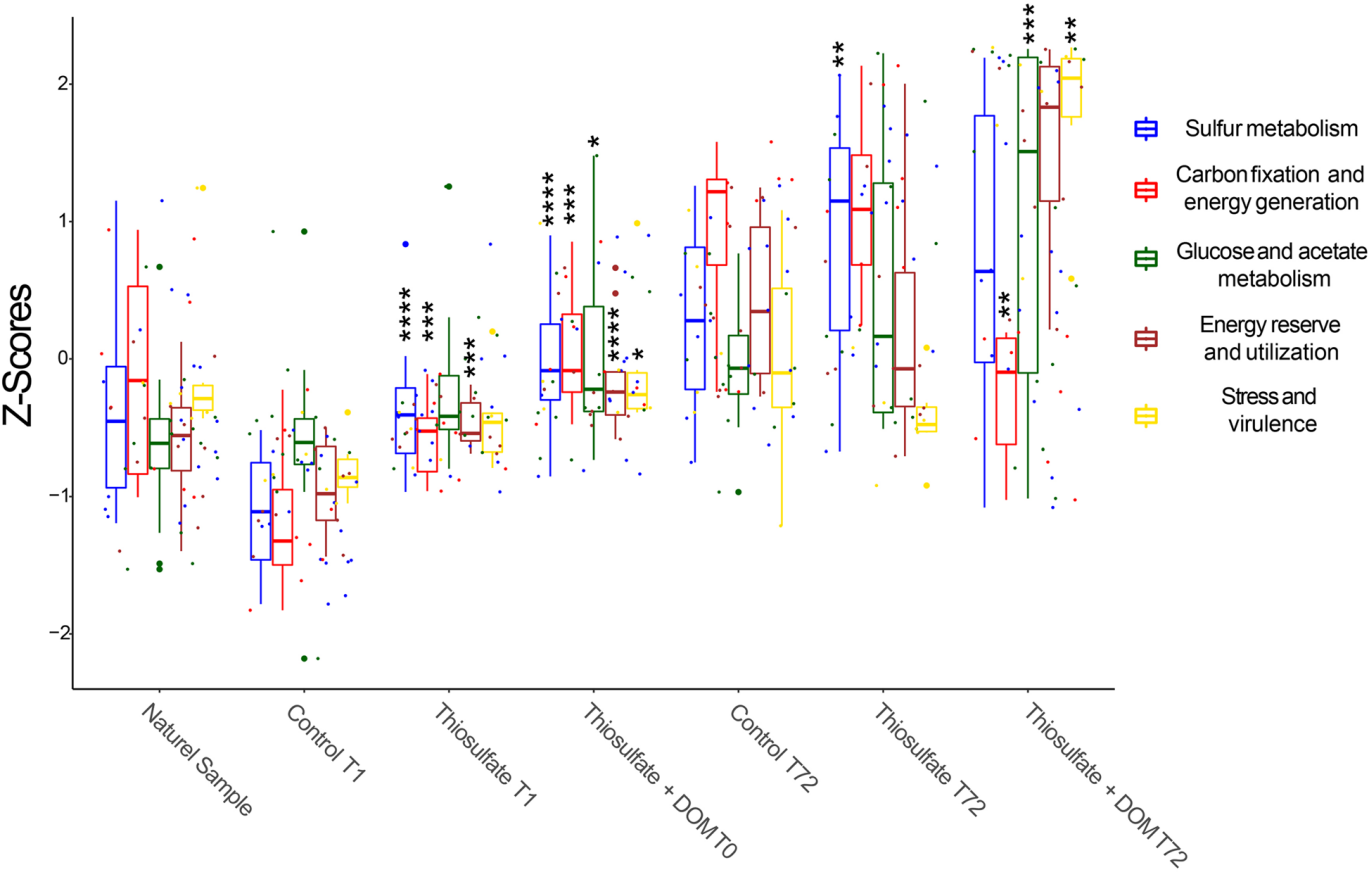
Comparison of z-scores between five selected metabolic categories depicted by color codings, i.e., blue, red, green, maroon, and yellow. Box plot showing the distribution of relative transcript expression data and their interquartile ranges in each sample. Median value of all transcripts expression in each sample is depicted in the form of a horizontal line in every box plot. Significance is indicated by asterisks, i.e., control at T1 and T72 *versus* treatments

Thiosulfate amendment, alone or combined with DOM, stimulated the transcription of phospholipid biosynthesis pathways genes up to twofold (Fig. 2, Table S2), while the availability of DOM additionally stimulated the synthesis of glycogen as an energy reserve (Figs. 2 and 3, Table S2). Additionally, the gene encoding the enzyme responsible for the biosynthesis of the sulfur-containing amino acid cysteine (cysteine synthase-encoding gene *cysK*) was threefold upregulated in the thiosulfate + DOM treatment compared to the unamended communities after 72 h (Fig. 2, Table S2). The assimilation of sulfate and thiosulfate generally leads to the biosynthesis of sulfur-containing amino acid [62]. Genes involved in glutathione (sulfur-containing antioxidant) biosynthesis were weakly upregulated (1.5-fold) in the thiosulfate amended treatment and up to 5.5-fold in the thiosulfate + DOM amendment (Fig. 2, Table S2). The respiratory chain can contribute up to 87% of the total intrinsic H_2_O_2_ production in aerobic bacteria [63]. Oxidation of glucose [64] and acetate [65] can contribute to the H_2_O_2_ production in bacteria [66]. Thus, elevated glutathione biosynthesis in the DOM-amended communities may help the cells to adapt to oxidative stress scenarios [67]. Additionally, superoxide dismutase-Fe (*sod*) and glutathione reductase (*gr*) transcripts were 1.5-fold and 2.4-fold upregulated, respectively, in the thiosulfate + DOM treatment as compared to unamended communities (Fig. 2A, Table S2). A 2, 3, and ninefold enrichment of cysteine-based peroxidase encoding *ahpC* and *tpx* genes and cysteine exporter (*cyd*C), respectively, were observed in the thiosulfate + DOM treatment compared to the unamended control, suggesting an important role of this amino acid in the detoxification of H_2_O_2_ as previously reported [68]. Cyclopropanate membrane lipids can also participate in the cell´s response against oxidative stress and extremely acidic pH conditions [69]. The prokaryotic community amended with thiosulfate + DOM was 1.3-fold enriched in cyclopropane-fatty-acyl-phospholipid synthase (*cfas*) transcripts compared to the unamended community (Fig. 2, Table S2). Acetate is not only used to synthesize acetyl-coA in the central metabolism, but it can also dissociate, generating protons or disturb the cellular osmolarity, and causing an acidic environment [70]. Consequently, during acetate-induced stress, cells may change the membrane permeability by stimulating the synthesis of specific fatty acids, such as the above-mentioned cyclopropane fatty acids. Moreover, the σ^38^ transcription factor that controls *cfas* gene transcription during stress [71] was also up-regulated in the thiosulfate + DOM amended community (Table S2). Thus, anti-stress factor-related genes were up-regulated in the thiosulfate + DOM treatment (the *z*-score value of the ‘stress and virulence’ category was twofold the *z*-score of the other samples, Fig. 3).

**Fig. 4:**
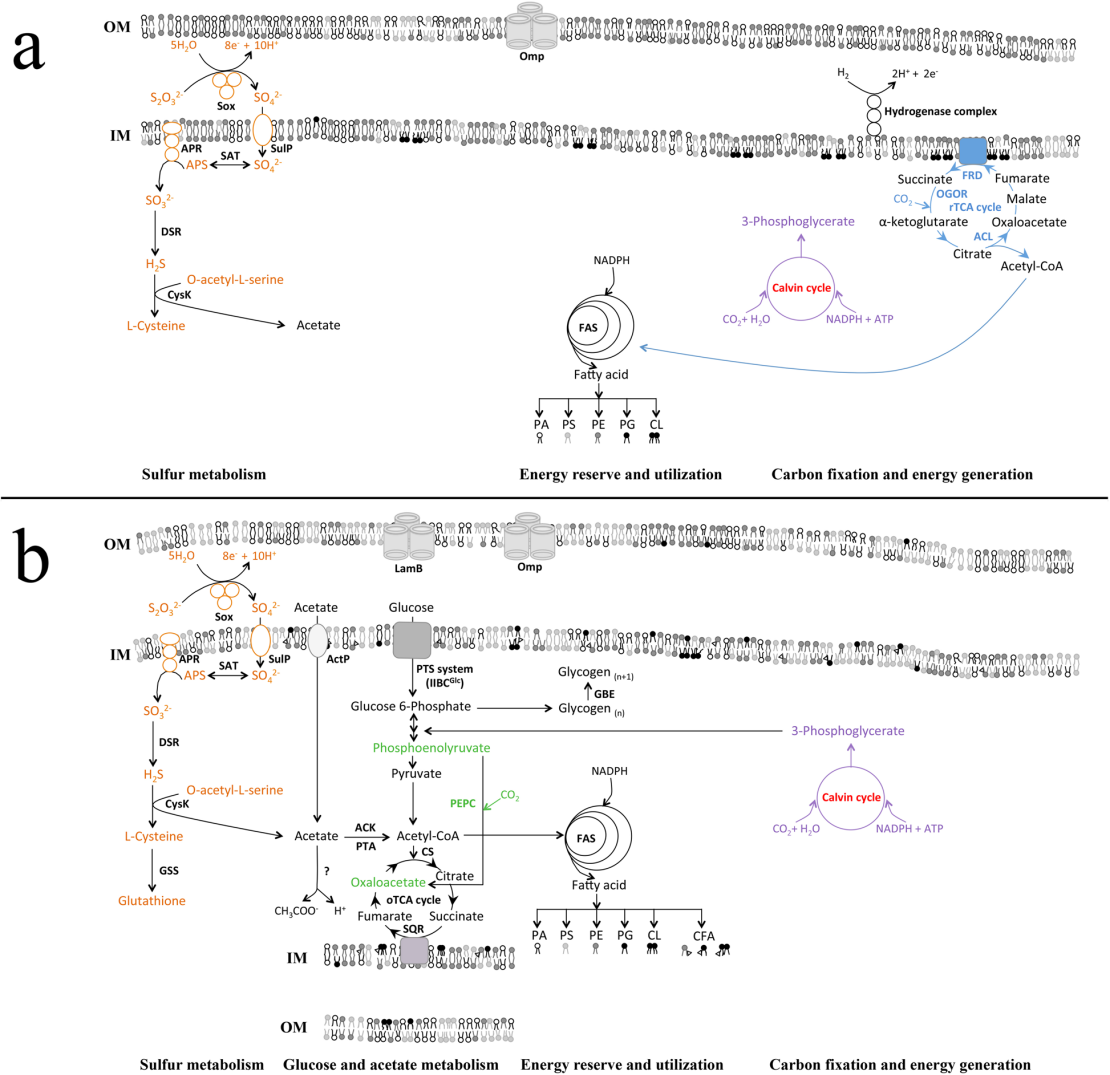
Summary of key metabolic processes in selected bathypelagic prokaryotic communities amended with **A** thiosulfate and **B** thiosulfate + DOM. Red font indicates lower transcript abundance in the amended communities as compared to unamended communities. Metabolic pathways drawn in brown belong to the sulfur utilization pathways. Blue plotted pathways are part of the reductive TCA (tricarboxylic acid) cycle. Purple is used to depict biochemical reactions from the Calvin Benson cycle. Green is used to represent reactions involved in the anaplerotic carbon fixation. Gene abbreviations: *ACK* Acetate kinase, *ACL* ATP citrate lyase, *ActP* Acetate permease, *Apr* Adenylylsulfate reductase, *CFA* Cyclopropane fatty acid, *CL* Cardiolipin, *CS* Citrate synthase, *CysK* Cysteine synthase, *DSR* Dissimilatory-type sulfite reductase, *FAS* Fatty acid biosynthesis, *FRD* Fumarate reductase, *GBE* Glycogen branching enzyme, *GSS* Glutathione synthetase, *IM* Inner membrane, *LamB* Maltoporin, *OGOR* 2-Oxoglutarate:ferredoxin oxidoreductase, *OM* Outer membrane, *OMP* Outer membrane protein, *oTCA cycle* Oxidative tricarboxylic acid cycle, *PA* Phosphatidic acid, *PE* Phosphatidylethanolamine, *PEPC* Phosphoenolpyruvate carboxylase, *PG* Phosphatidylglycerol, *PS* Phosphatidylserine, *PTA* Phosphate acetyltransferase, *PTS* system, glucose-specific IIBC component, *rTCA* Reductive tricarboxylic acid cycle, *SAT* Sulfate adenylyltransferase, *Sox* Sulfur oxidation protein, *SQR* Succinate-ubiquinone oxidoreductase, *SulP* Sulfate transporter

Overall, an episodic availability of energy sources not only promotes and temporarily sustains microbial communities but also supports energy storage (Fig. 4). Figure 4 summarises our observations where thiosulfate amendment resulted in an up-regulation of the electrogenic multi-enzyme Sox pathway and hydrogenase complex system. A stimulation of sulfur oxidation also promoted sulfur-containing amino acid biosynthesis. Autotrophic CO_2_ fixation pathway genes of the reductive TCA and Calvin cycle were detected, the latter pathway was not up-regulated. however. Whether acetyl-CoA resulting from the reductive TCA cycle can fuel fatty acid biosynthetic pathway requires detailed experiments that were not conducted in this study.

Acetate and glucose as carbon sources were imported by respective transporters, i.e., the ActP and PTS system (Fig. 4). Major outer membrane protein (Omp) and maltoporin (LamB) genes were upregulated in the thiosulfate + DOM treatment. Glucose may get imported into the periplasmic space via Omp or LamB, however, it is the phosphoenolpyruvate (PEP)-sugar phosphotransferase (PTS) system that controls the preferential transport of glucose over other carbon sources. Thiosulfate + DOM amendment stimulated glycogen biosynthesis transcripts (Fig. 2A). Thiosulfate + DOM amendment also promoted the enrichment of *pepc* transcripts. Phosphoenolpyruvate carboxylase enrichment points to the importance of the anaplerotic reactions of DIC fixation, which can also replenish Krebs cycle intermediates in the thiosulfate + DOM treatment as compared to the thiosulfate amendment.

Acetate utilization is controlled by the PTA-ACK (phosphotransacetylase—acetate kinase) pathway (Fig. 4). The protonated form of acetate can easily diffuse across the cell membrane; however, the inner membrane contains cation/acetate symporter ActP selectively transporting acetate into the cell. Acetyl-CoA originating from glucose or acetate not only generates energy by operating the oxidative TCA cycle but also participates in fatty acid biosynthesis (that also produces energy by releasing ATP upon catabolism). In fact, phospholipid biosynthetic pathways were stimulated in both amendments, suggesting that this type of carbon/energy storage is common in chemoautotrophs and heterotrophs. Thiosulfate + DOM-amendment additionally promoted overexpression of the cyclopropane fatty acid-encoding gene. Calvin cycle genes that were less represented in our experiments compared to the control samples should produce 3-phosphoglycerate that can be channeled into the glycolytic pathway.

## Conclusions

Bathypelagic prokaryotic communities responded to thiosulfate addition by increased metabolic activity and expression of genes supporting energy production. However, the response varied based on whether DOM was also added. While chemoautotrophic DIC fixation was stimulated after the addition of thiosulfate, the availability of DOM stimulated anaplerotic DIC fixation. Taken together, our results point to the potential not only for chemoautotrophy but also for anaplerotic DIC fixation in deep-sea prokaryotes, particularly in nutrient-rich microenvironments such as marine snow, where thiosulfate, as well as organic matter, might be readily available. Additionally, the results suggest the interconnectivity between auto- and heterotrophic cells through the release of organic and inorganic metabolites.

### Supplementary Information

**Additional file 1: Figure S1.** A natural prokaryotic community collected from 2000 m depth in the North Atlantic.

**Additional file 2: Table S1. **Physicochemical and biological characteristics of the LSW at the sampling location.

**Additional file 3: Table S2. **Normalized transcript abundance from microbial communities in non-amended (control) and amended (Thiosulfate, Thiosulfate+DOM) communities.

**Additional file 4: Table S3a. **Relative taxonomic abundance of microbial communities expressing genes (presented here in form of KOs or KEGG identifiers) in non-amended (control) water sample after one hour incubation in the dark at in situ temperature i.e. T1.** Table S3b. **Relative taxonomic abundance of microbial communities expressing genes (presented here in form of KOs or KEGG identifiers) in thiosulfate amended water sample after one hour incubation in the dark at in situ temperature i.e. T1.** Table S3c. **Relative taxonomic abundance of microbial communities expressing genes (presented here in form of KOs or KEGG identifiers) in Thiosulfate+DOM amended water sample after one hour incubation in the dark at in situ temperature i.e. T1.

**Additional file 5. **Supplementary text.

## Data Availability

The GEO accession ID is GSE136729 including the seven metatranscriptome datasets (GSM4056809-GSM4056815).
