# Supplementary figures and images for: Interplay between autotrophic and heterotrophic prokaryotic metabolism in the bathypelagic realm revealed by metatranscriptomic analyses
Source: Microbiome. 2023 Nov 4;11:239. doi: 10.1186/s40168-023-01688-7 (PMC10625248; doi:10.1186/s40168-023-01688-7)

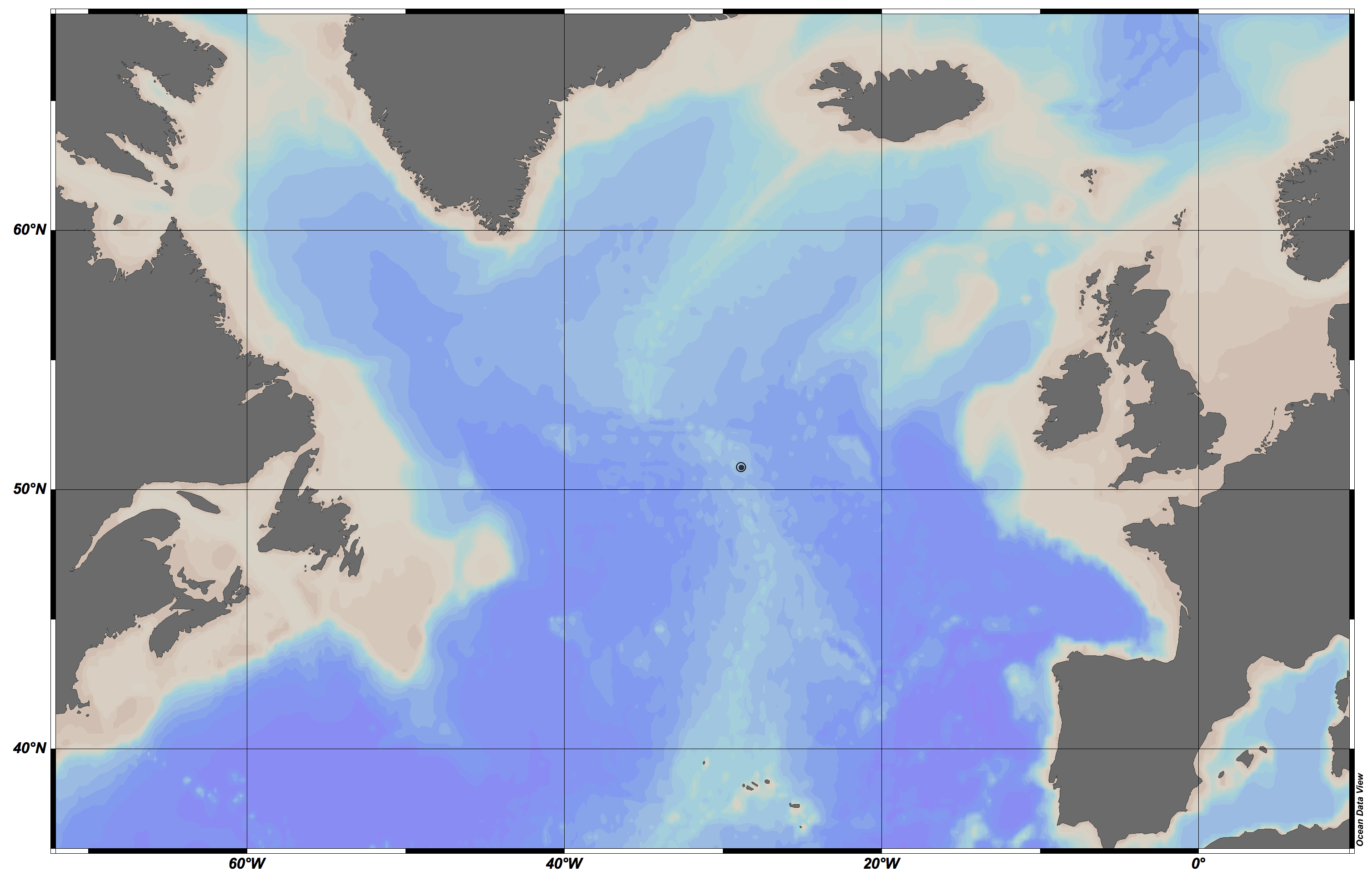

Supplement: Supplementary file 2 — Additional file 1: Figure S1. A natural prokaryotic community collected from 2000 m depth in the North Atlantic. [file 40168_2023_1688_MOESM1_ESM.jpg]
