# Supplementary material for: Interplay between autotrophic and heterotrophic prokaryotic metabolism in the bathypelagic realm revealed by metatranscriptomic analyses
Source: Microbiome. 2023 Nov 4;11:239. doi: 10.1186/s40168-023-01688-7 (PMC10625248; doi:10.1186/s40168-023-01688-7)
Supplement: Supplementary file 6 — Additional file 5. Supplementary text. [file 40168_2023_1688_MOESM5_ESM.pdf]

Supplementary text

Interplay between autotrophic and heterotrophic prokaryotic metabolism in the bathypelagic realm revealed by metatranscriptomic analyses

Abhishek Srivastava[1,9*], Daniele De Corte[2,7], Juan AL Garcia[1,8], Brandon K. Swan[3], Ramunas Stepanauskas[4], Gerhard J. Herndl[1,5], Eva Sintes[6#]

[1] Department of Functional and Evolutionary Ecology, Bio-Oceanography and Marine Biology Unit, University of Vienna, Djerassiplatz 1, 1030 Vienna, Austria

[2] Institute for Chemistry and Biology of the Marine Environment, Carl Von Ossietzky University, Oldenburg, Germany

[3] National Biodefense Analysis and Countermeasures Center, Frederick, MD 21702, USA

[4] Bigelow Laboratory for Ocean Sciences, East Boothbay, ME 04544, USA

[5] NIOZ, Department of Marine Microbiology and Biogeochemistry, Royal Netherlands Institute for Sea Research, AB Den Burg, The Netherlands

[6] Ecosystem Oceanography Group (GRECO), Instituto Español de Oceanografía (IEO-CSIC), Centro Oceanográfico de Baleares, Palma, Spain

[7] Currently at Ocean Technology and Engineering Department, National Oceanography Centre, Southampton, UK

[8] Department of Informatics, INS La Ferreria, 08110 Montcada i Reixach, Spain

[9] Konrad Lorenz Institute of Ethology, University of Veterinary Medicine Vienna, Savoyenstrasse 1a, 1160 Vienna, Austria

* Co-corresponding author email: abhishek.srivastava@univie.ac.at
# Corresponding author email: Eva.Sintes@ieo.csic.es

Short title: Metatranscriptomic analyses of bathypelagic waters

Supplementary highlights of other interesting metabolic features in metatranscriptomes data

In order to incorporate nitrogen-based nutrients, amino acid transporter subunits-encoding transcripts were upregulated subtle in thiosulfate alone treatment and strongly in thiosulfate+DOM amendment (Table S2). Importantly, the L-amino acid transporter AapJQMP can both, import and export amino acids [1]. Upregulation of this feature suggests not only microbial self-nourishment but also community sustenance. Transcription of leucyl aminopeptidase-encoding gene (*pepA*) and of the leucine-responsive regulatory protein-encoding gene (*lrp*) was upregulated by 1.6-fold and nearly five-fold, respectively, in the thiosulfate+DOM treatment (Table S2). Lrp is a global transcriptional regulator that detects the environmental nutritional status and regulates the expression of genes involved in various metabolic functions, including nutrient transport and motility [2]. Interestingly, *lrp* was also two-fold upregulated in the thiosulfate-amended treatment. Furthermore, the genes encoding the exo- and/or ectoenzymes lipase, thermolabile hemolysin, alkaline phosphatase, alpha-amylase, beta-glucosidase and leucyl aminopeptidase (Fig. 2, Table S2) were upregulated (1.5- to 54-fold compared to the control) in the thiosulfate+DOM amended communities and were putatively assigned to members of Gammaproteobacteria (*Vibrionales* and *Pseudoalteromonas*).

Flagella-related gene transcription was stimulated in both thiosulfate and the thiosulfate+DOM amended communities (Table S2). However, chemotaxis and quorum sensing were only overexpressed in the latter amendment (Table S2). Flagella encoding transcripts were mainly assigned to Gammaproteobacteria followed by Deltaproteobacteria, Firmicutes and Alphaproteobacteria members (Table S3). Transcripts of quorum-sensing signal molecule biosynthesis (*luxS*) was 24-fold enriched in the thiosulfate+DOM amended community as compared to the unamended control (Table S2). This suggests that the promotion of bacterial communication based upon autoinducer-2. *luxS* transcripts was associated with Gammproteobacteria (~82%), Epsilonproteobacteria (13%) and unclassified bacteria (~5%) (Table S3). Glucose was previously shown to promote quorum sensing by the overexpression of autoinducer-2 [3].

The upregulation of genes related to motility, chemotaxis and quorum sensing in the thiosulfate and thiosulfate+DOM amendments indicates an active response of the

members of the community to gradients in these substrates that is possibly facilitated by cell-to-cell communications.

References

1. Walshaw DL, Poole PS. The general L-amino acid permease of *Rhizobium leguminosarum* is an ABC uptake system that also influences efflux of solutes. Mol Microbiol. 1996;21:1239-1252.
2. Ziegler CA, Freddolino PL. The leucine-responsive regulatory proteins/feast-famine regulatory proteins: an ancient and complex class of transcriptional regulators in bacteria and archaea. Crit Rev Biochem Mol Biol. 2021;56:373-400.
3. Cloak OM, Solow BT, Briggs CE, Chen CY, Fratamico PM. Quorum sensing and production of autoinducer-2 in *Campylobacter* spp., *Escherichia coli* O157:H7, and *Salmonella enterica* serovar Typhimurium in foods. Appl Environ Microbiol. 2002;68:4666-4671.
